# Variability of Sleep Duration Is Related to Subjective Sleep Quality and Subjective Well-Being: An Actigraphy Study

**DOI:** 10.1371/journal.pone.0071292

**Published:** 2013-08-14

**Authors:** Sakari Lemola, Thomas Ledermann, Elliot M. Friedman

**Affiliations:** 1 Department of Psychology, University of Basel, Basel, Switzerland; 2 Human Development and Family Studies, Purdue University, West Lafayette, Indiana, United States of America; University of Alabama at Birmingham, United States of America

## Abstract

While there is a large body of evidence that poor subjective sleep quality is related to lower subjective well-being, studies on the relation of objective sleep measures and subjective well-being are fewer in number and less consistent in their findings. Using data of the Survey of Mid-Life in the United States (MIDUS), we investigated whether duration and quality of sleep, assessed by actigraphy, were related to subjective well-being and whether this relationship was mediated by subjective sleep quality. Three hundred and thirteen mainly white American individuals from the general population and 128 urban-dwelling African American individuals between 35 and 85 years of age were studied cross-sectionally. Sleep duration, variability of sleep duration, sleep onset latency, and time awake after sleep onset were assessed by actigraphy over a period of 7 days. Subjective sleep quality was assessed with the Pittsburgh Sleep Quality Index, positive psychological well-being and symptoms of psychological distress were assessed with the Satisfaction with Life Scale and the Mood and Anxiety Symptom Questionnaire. In both white and African Americans high day-to-day variability in sleep duration was related to lower levels of subjective well-being controlling age, gender, educational and marital status, and BMI. By contrast, sleep duration, sleep onset latency, and time awake after sleep onset were not related to subjective well-being controlling covariates and other sleep variables. Moreover, the relationship between variability in sleep duration and well-being was partially mediated by subjective sleep quality. The findings show that great day-to-day variability in sleep duration – more than average sleep duration – is related to poor subjective sleep quality and poor subjective well-being.

## Introduction

Subjective well-being, a construct that includes general satisfaction with life and positive affect [1], [2], is widely linked to better health and greater longevity [3], [4], and there is ongoing interest in the factors that predict well-being [1], [5]. Recent studies suggest that poor sleep [6]–[12] can impair well-being, but most of these studies have involved individuals’ perceptions of sleep quality and duration; only a few studies have examined associations with objectively-assessed sleep. Such assessments are important given well-documented dissociations between subjective reports of sleep quality and objective sleep measurement [13]–[15]. The current study uses both subjective and objective assessments of sleep in two samples of middle-aged and older adults to examine links to well-being as well as the degree to which subjective perceptions of sleep mediate the relationship between objective sleep measures and well-being. Importantly, the focus in this study is on both intra-individual variability in sleep as well as overall averages of sleep duration and quality, a relatively new focus for population level sleep research.

The relation of self-reported sleep duration and subjective sleep quality with well-being has repeatedly been shown during the last 15 years. Individuals who reported poor sleep quality and short sleep duration were consistently lower in subjective well-being and also reported increased levels of negative affect and mood disturbances when compared to individuals who reported good sleep quality and/or 7–8 hours of sleep per night [6]–[12]. In contrast, only few studies have addressed the relationship between objective measures of sleep and subjective well-being and their overall findings have been less consistent. Ryff and coworkers [16] found that sleep duration, as assessed with the Nightcap system that monitors eyelid motion, was positively related with measures of psychological well-being in women above the age of 65 years. However, Jean-Louis and coworkers [15] found no relationship between actigraphic assessments of sleep duration, sleep onset latency, and sleep efficiency with subjective well-being in an adult general population sample. Similarly, Mezick et al. [17], using actigraphy data from the Pittsburgh SleepSCORE study including middle aged and older individuals, found no relationship between sleep duration (and fragmentation) and reports of stressful life events. In adults meeting diagnostic criteria for chronic insomnia there was only a weak link between sleep duration and sleep efficiency measured with polysomnography and daytime fatigue [18]. On the other hand, reports of daytime fatigue were more strongly related to poor subjective sleep quality and poor mental health in this study.

In sum, self-reported sleep measures are more consistently related to varied assessments of subjective well-being than are objective sleep assessments. One explanation for the generally strong relation between self-reported sleep and subjective well-being (which is not reflected when objective measures of sleep are taken into account) involves common method bias. The valence of self-reports can be co-linear across domains – individuals with a negativity bias in their self-reports in one domain may show the same negativity bias in other domains [19]. This interpretation is also in line with the finding that subjective sleep ratings and objective measures of sleep quality often show a relatively modest or even non-existent relationship [13]–[15]. Baker et al. [13] reported no significant relation between polysomnographic derived sleep onset latency and number of awakenings measured during three nights of observation and subjective ratings of sleep quality in 20 healthy individuals. Similarly, Jean-Louis et al. [15] reported that sleep efficiency, assessed by actigraphy over three nights, was unrelated to general sleep satisfaction in a study with 273 healthy adults. A positive correlation of.24 (*P*<.003) between polysomnographic derived sleep efficiency measured during one night and general subjective sleep quality in healthy older individuals was found by Vitiello et al. [14]. There were important limitations to all these studies, including basing estimates of average sleep parameters on a small number of nights of polysomnography or actigraphy [14], [15] or relatively small sample size with consequently low statistical power to detect associations [13]. Notwithstanding these limitations, Sadeh [20] recently concluded that the correspondence of actigraphy measures of sleep quality (night-wakings, wake after sleep onset, sleep efficiency) with subjective reports of sleep quality is relatively poor, although it remains unclear to what extent such discrepancies should be attributed to inaccuracy in actigraphy versus inaccuracy of subjective reporting.

An alternative explanation of this discrepancy is that other sleep parameters that are less commonly reported, such as variability in sleep duration across multiple nights, may contribute to an individual’s perceptions of sleep quality and subjective well-being. Typically, studies that have examined variability in sleep have reported larger within-person variability of sleep duration across days than between-person variability of sleep duration [21], [22]. Moreover, studying intra-individual variability of sleep duration is important as it may contribute to sleep problems [23]–[28]. For instance, individuals with insomnia have more variable sleep times than healthy people [24], [28], and it is thought that inconsistent sleep patterns may be a mechanism by which insomnia is sustained. Individuals who sleep poorly on one night may want to ‘catch up’ by sleeping longer during the subsequent night. Although they– possibly due to increased homeostatic sleep pressure– may succeed in getting relief by ‘recovery’ sleep, this can even result in worse sleep during the following night, as overly long sleep may further undermine the actual ability to fall asleep [29], [30]. Therefore, establishing a regular sleep–wake schedule is an integral part of behavioral treatment of insomnia along with reduction of time spent in bed, stimulus control therapy, and sleep hygiene instructions [31].

Importantly, variability in sleep duration is linked to both perception of sleep quality and subjective well-being. In healthy adults who maintained sleep diaries for two weeks, irregular patterns of activity and sleep were strongly related to lower subjective sleep quality [32]. Mezick et al. [17] found that negative affect and reports of stressful life events were related with both variability of sleep duration and variability of sleep fragmentation as assessed by actigraphy. Scores of variability in sleep duration and fragmentation were particularly increased in individuals who experienced both high stress levels and high levels of negative affect [17]. In adolescents, a large weekday to weekend difference in sleep duration was related to increased vulnerability towards the negative effects of parental conflict [33]. Moreover, adolescents with higher intra-individual day-to-day variability of sleep duration assessed by sleep log over a period of two weeks was related with depression, anxiety, and fatigue controlling average sleep duration [7]. Finally, in older adults suffering from dementia, individuals with more regular sleep-wake patterns assessed by actigraphy had better subjective well-being and daily functioning, and less cognitive decline [34], [35].

A first aim of the present study is to test the relationship between actigraphy defined sleep duration and sleep quality with subjective well-being. In addition to the average sleep duration, we include intra-individual variability of sleep duration across seven consecutive nights of actigraphy assessment. We hypothesized that intra-individual variability of sleep duration would explain variance of subjective well-being beyond what can be explained by average measures of sleep indices.

As a second aim we test a mediation model, which postulates that subjective sleep quality is a mediator of the relationship between the objective measures of sleep and subjective well-being. We test this mediation model based on the theoretical assumption that subjective sleep quality is to some degree a reflection of objectively measurable sleep quality (although empirical evidence for this assumption has been mixed; [13]–[15], [20]) and that objectively measured sleep quality has an impact on well-being through subjectively perceived sleep quality.

As there is evidence that African American individuals on average report lower levels of sleep quality, shorter sleep duration, and more variability of sleep duration than white individuals [17], [22], [36], [37], [38] we tested whether race(/residential neighborhood) moderated the relationship between actigraphy sleep indices and subjective well-being by assessing differences in these relations in a sample of predominantly white individuals and a sample of African Americans from a highly segregated urban neighborhood.

## Methods

### Participants and Procedure

The data for this study come from the MIDUS II (Midlife in the United States) bioindicator study (see for details on recruitment reference [39]). Actigraphic sleep assessment was conducted with 441 participants who attended the MIDUS Research Center at the University of Wisconsin–Madison. Among these, 313 participants were recruited by random digit dialing or from a national twin registry (93.9% were Caucasian); we here refer to this subsample as the “predominantly white American sample”. Furthermore, 128 African American adults were from an economically less affluent urban neighborhood in Milwaukee County with at least 40% Black residents. Milwaukee is considered as one of most highly segregated cities in the United States [40]. We here refer to this subsample as the “urban African American sample”. The sample characteristics are presented in Table 1. The study protocol was approved by the Social and Behavioral Science Institutional Review Board of the University of Wisconsin-Madison. Written informed consent was obtained from each participant.

**Table 1:** Sample characteristics and reliabilities, means, and standard deviations of study variables in both subsamples.

	Predominantly White subsample(*n* = 313)			African American subsample(*n* = 128)			
	*α*	*M*/%	*SD*	*α*	*M*/%	*SD*	*F* a/*Chi* ^2^ a
Age (years)	–	57.65	11.80	–	54.89	10.28	5.34*
Gender (% female)	–	55.3%		–	72.7%		11.46***
Education (% high school/General Education Diploma or less)	–	26.9%		–	44.5%		12.92***
Marital status (% married)	–	71.9%		–	26.6%		76.99***
BMI	–	29.47	5.90	–	33.41	9.33	28.28***
Total Sleep Time (min)	.86	381.60	62.39	.80	339.00	72.72	38.36***
Variability of Total Sleep Time	–	15.39	8.81	–	23.37	14.66	49.35***
Wake After Sleep Onset (min)	.85	44.35	19.95	.70	61.19	29.86	47.60***
Sleep Efficiency (%)	.91	82.15	8.56	.87	71.95	11.51	104.47***
Sleep Onset Latency (min)	.77	25.15	21.97	.74	47.37	44.23	49.36***
PSQI: Poor subjective sleep quality	.77	5.81	3.37	.74	8.08	4.42	29.89***
Satisfaction with Life Scale	.89	4.96	1.27	.78	4.14	1.22	38.22***
Positive Affect	.94	44.80	10.46	.91	43.47	10.62	1.45
General Distress Depressive Sympt.	.89	18.14	6.14	.92	20.37	8.39	9.51**
General Distress Anxious Sympt.	.78	16.22	4.28	.91	17.51	6.83	5.70*
Loss of Interest	.77	11.74	3.84	.87	13.27	5.65	10.83**
Anxious Arousal	.73	21.05	4.24	.85	23.95	7.24	27.17***

*Note*. ***α*** = Cronbach’s ***α***.

a comparison of mean values/frequencies between participants from the predominantly white American sample and the urban African American sample. Abbreviations: PSQI: Pittsburgh Sleep Quality Index.

* *P*<.05,

** *P*<.01,

*** *P*<.001.

### Measurement

#### Positive well-being

Positive well-being was measured by two scales, one reflecting satisfaction with life and the other one reflecting positive affect.

Satisfaction with Life. The Satisfaction with Life Scale (SWLS; [41]) was used to assess individuals’ evaluations of their life in general. Five items (e.g., “I am satisfied with my life”) were rated on a seven-point scale (1 = Strongly disagree; 7 = Strongly agree) and combined together so that higher scores indicate higher well-being. Information on descriptive statistics and reliability of the scales is presented in Table 1.

Positive Affect. Positive affect was assessed using the High Positive Affect subscale from the Mood and Anxiety Symptom Questionnaire (MASQ; [42]). Participants were asked to report how much they felt 14 distinct positive mood states during the past week on a five-point scale (1 = not at all; 5 = extremely). A sample item includes “I felt cheerful”. The items were combined together so that higher scores reflect more positive affect.

### Symptoms of Distress

To assess symptoms of distress we used four subscales from the MASQ (General Distress Depressive Symptoms subscale, the General Distress Anxious Symptoms subscale, the Loss of Interest subscale, and the Anxious Arousal subscale; [42]). In the MASQ-scales participants were asked to report how much they felt different symptoms of distress during the past week using the same five-point scale as for the positive affect subscale. The General Distress Depressive Symptoms subscale includes 12 items measuring feelings of depression such as “felt sad”. The General Distress Anxious Symptoms subscale includes 11 items measuring feelings of anxiety and uneasiness such as “felt afraid”. The Loss of Interest subscale includes 8 items measuring lack of energy and interest such as “felt nothing was very enjoyable”. The Anxious Arousal subscale includes 17 items measuring bodily sensations indicating anxiety and distress such as “heart was racing or pounding”.

### Subjective Sleep Quality

Subjective sleep quality was assessed using the Pittsburgh Sleep Quality Index (PSQI; [43]) which includes seven subscales (subjective sleep satisfaction, sleep latency, sleep duration, habitual sleep efficiency, sleep disturbance, use of sleep medication, and daytime dysfunction) that are combined into a global score ranging from 0–21. The global score for the PSQI was available for 399 participants (291 in the predominantly white American sample and 108 in the urban African American sample).

### Actigraphy

Detailed descriptions of actigraphy data collection and scoring methods are publicly available at the Interuniversity Consortium for Political and Social Research (ICPSR) website (URL: http://www.icpsr.umich.edu). Briefly, the Mini Mitter Actiwatch®-64 activity monitor was used to detect the number of movements with a built-in motion sensor. The devices were worn on the non-dominant wrist for seven consecutive days and nights and programmed to begin collecting data at 7∶00 a.m. on the Tuesday following the visit at the study center and to conclude the following Tuesday morning. Participants completed daily diaries in which they indicated bed time and rise time, and these were used as start and end times for the actigraphic records. In some cases (after the technology became available in 2005), participants also used an event marker on the actiwatch to indicate bed and rise times. These markers were used in 28 cases when diary entries were missing for a specific day (and the markers appeared reliable based on the actigraphic record). The participants returned the Actiwatch® by postage paid envelope and data was downloaded from the Actiwatch® and stored in the Actiwatch® database for processing upon receipt in the project office. If participants provided incomplete information (e.g., if they forgot to put the watch on or took it off too early etc.) or had exceptional experiences during the data collection period (e.g. travel to a different time zone), which made scoring difficult, the cases were reviewed and the intervals were deleted.

The Actiware 5 software and manufacturer algorithms for detecting sleep based on 30 sec epochs were used to generate summary statistics about the participants’ sleep. In detail, whether a particular epoch was scored as wake or sleep was determined by comparing activity counts for the epoch in question and those immediately surrounding it to a threshold value following the formula validated by Oakley (1997 [44]). A medium-sensitivity threshold was used for data evaluation. The actigraphy sleep indices that were derived include total sleep time, sleep onset latency, wake after sleep onset, and sleep efficiency. Day-to-day variability of sleep duration was calculated as the mean referenced variation (individual standard deviation of sleep duration across the 7 days of measurement divided by the individual average of sleep duration×100; coefficient of variation, see e.g., [45]). According to Rowe and coworkers [45] 7 days of measurement are sufficient to derive a reliable estimate of the variability of sleep duration. Because of skewness, values for day-to-day variability of sleep duration were log-transformed for analyses. Of the 441 participants who took part on the actigraphy protocol 398 (90.2%) had measurements of all 7 nights, 24 (5.4%) had measurements of 6 nights, 5 (1.1%) had measurements of 5 nights, 9 (2.0%) had measurements of 4 nights, and 5 (1.1%) had measurements of 3 nights.

### Statistical Analyses

First, product-moment correlations were calculated to assess the interrelationships among the sleep measures in the combined sample as well as in the two subsamples (the predominantly white American sample and the urban African American sample), separately.

Second, multiple regressions were conducted to predict indices of positive well-being and symptoms of distress by total sleep time, day-to-day variability of total sleep time, sleep onset latency, and wake after sleep onset that were simultaneously entered into the models. Sleep efficiency showed almost complete overlap with total sleep time, sleep onset latency, and wake after sleep onset (*R* = .95, *P*<.001, when predicting sleep efficiency by total sleep time, sleep onset latency, and wake after sleep onset in a multiple regression model) and was therefore not included as predictor in the multiple regressions. To test differences between the two subsamples (predominantly white American sample vs. urban African American sample) regarding the associations of actigraphy sleep indices with indices of subjective well-being, product interaction terms (sample membership coded 0 and 1 multiplied with centered actigraphy sleep indices) were entered in an additional step (see [46] ). Additional models were calculated including the quadratic term of total sleep time controlling for the linear term of total sleep time as evidence indicates that short as well as long sleep duration might relate to worse outcomes [6], [9]. Before building the quadratic term, total sleep time was standardized. In order to minimize type I error inflation, Bonferroni correction was applied and statistical significance level set at *P*<.0083 (i.e., by dividing the standard significance level of *P*<0.05 by 6, as six measures of subjective well-being were used as dependent variables).

Third, structural equation modeling with Maximum Likelihood estimation and AMOS 18 [47] was used to test a mediation model with subjective sleep quality (assessed by the PSQI) as the mediator of the relationship of actigraphic total sleep time, day-to-day variability of total sleep time, sleep onset latency, and wake after sleep onset with subjective well-being. The latent construct of subjective well-being was measured with two indicators reflecting positive well-being and symptoms of distress, respectively. The indicator of positive well-being was built by averaging the standardized scores of Satisfaction with Life and the MASQ-positive affect which were highly correlated (*r* = .47; *P*<.001). The indicator of symptoms of distress was built by averaging the standardized scores of the MASQ subscales of General Distress Depressive Symptoms, General Distress Anxious Symptoms, Loss of Interest, and Anxious Arousal (*r*-Range = .58–.79). Models with a root mean square error of approximation (RMSEA) of.06 or less, a comparative fit index (CFI) of.95 or higher, a goodness of fit index (GFI), and an adjusted goodness of fit index (AGFI) of.90 and higher were considered as consistent with the data [48]. The statistical significance of the indirect effects of actigraphy measures on subjective well-being via subjective sleep quality as mediator was evaluated by calculating bootstrap confidence intervals using 1000 parametric bootstrap samples [49]. Multiple regression analyses and the structural equation model include participant gender, age, marital status, education, BMI, sample membership (predominantly white American sample vs. urban African American sample), and twin status (a dummy variable indicating whether a participant was from the MIDUS-twin sample) as covariates as these variables may be related to both subjective well-being and sleep [37], [50]–[53]. More detailed information on the procedure applied in model estimation can be found in supporting information (Table S1).

## Results

Table 1 shows the descriptive statistics for the study variables within the predominantly white American sample and the urban African American sample. The participants in the predominantly white American sample showed on average longer total sleep time (Cohen’s *d* = 0.63, *P*<0.001), lower day-to-day variability of sleep duration (*d* = −0.40, P<0.001), less wake after sleep onset (*d* = −0.66, *P*<0.001), higher sleep efficiency (*d* = 1.01, *P*<0.001), shorter sleep onset latency (*d* = −0.64, *P*<0.001), and better subjective sleep quality as assessed by the PSQI (*d* = −0.58, *P*<0.001) than the participants in the urban African American sample. Furthermore, the predominantly white American sample had substantially higher scores in satisfaction with life (*d* = 0.66, *P*<0.001), and lower scores in symptoms of distress (depressive symptoms: *d* = −0.30, *P* = 0.002; anxious symptoms: *d* = −0.23, *P* = 0.02; loss of interest: *d* = −0.32, *P* = 0.001; and anxious arousal: *d* = −0.49, *P*<0.001).

In both samples women had on average longer total sleep time than men (predominantly white American sample: *d* = 0.58, *P*<0.001; urban African American sample: *d* = 0.41, *P* = 0.03), they had higher sleep efficiency (predominantly white American sample: *d* = 0.64, *P*<0.001; urban African American sample: *d* = 0.65, *P* = 0.001), and shorter sleep onset latency (predominantly white American sample: *d* = −0.45, *P*<0.001; urban African American sample: *d* = −0.48, *P* = 0.01). In the predominantly white American sample, women were lower in wake after sleep onset (*d* = −0.34, *P* = 0.003) and they were higher in anxious arousal (*d* = 0.23, *P* = 0.04). Furthermore, women in the urban African American sample had lower day-to-day variability of sleep duration than men (*d* = −0.41, *P* = 0.04). No gender differences in both samples could be found for subjective sleep quality as assessed by the PSQI, satisfaction with life, positive affect, depressive symptom scores, anxious symptom scores, and loss of interest scores.

Table 2 shows product-moment correlations among actigraphy sleep variables and subjective sleep quality. Short total sleep time, higher variability of total sleep time, large sleep onset latency and wake after sleep onset, and low sleep efficiency were related to poor subjective sleep quality. In a (post-hoc) multiple regression model predicting subjective sleep quality by total sleep time, variability of total sleep time, wake after sleep onset, and sleep onset latency a significant portion of the variance of subjective sleep quality could be explained (in the combined sample: adjusted *r^2^ = 0.14*; *F*(4/398) = 17.16, *P*<0.001).

**Table 2:** Zero-order correlations between sleep indicators.

	Total SleepTime	Variability of TotalSleep Time	Wake After SleepOnset	SleepEfficiency	Sleep OnsetLatency
**Variability of Total Sleep Time**					
Combined sample	−.49***				
Predominantly White subsample	−.43***				
African American subsample	−.47***				
**Wake After Sleep Onset**					
Combined sample	−.09*	.25***			
Predominantly White subsample	−.06	.18**			
African American subsample	.07	.16			
**Sleep Efficiency**					
Combined sample	.61***	−.57***	−.65***		
Predominantly White subsample	.59***	−.51***	−.64***		
African American subsample	.52***	−.52***	−.56***		
**Sleep Onset Latency**					
Combined sample	−.35***	.43***	.38***	−.78***	
Predominantly White subsample	−.36***	.42***	.24***	−.74***	
African American subsample	−.24**	.36***	.37***	−.79***	
**PSQI: Poor subjective sleep quality**					
Combined sample	−.13*	.30***	.29***	−.30***	.25***
Predominantly White subsample	−.10	.29***	.18**	−.18**	.16**
African American subsample	.01	.17	.30***	−.26**	.23**

*Note*. Abbreviations: PSQI: Pittsburgh Sleep Quality Index.

* *P*<.05,

** *P*<.01,

*** *P*<.001.

Multiple regressions predicting measures of positive well-being and symptoms of distress by total sleep time (linear and curvilinear effects), variability of total sleep time, sleep onset latency, and wake after sleep onset are presented in Table 3 for the combined sample as well as the subsamples. In the combined sample, higher variability of total sleep time was predictive of lower satisfaction with life, higher depressive symptom scores, anxious symptom scores, loss of interest scores, and anxious arousal scores at the Bonferroni-corrected significance level of *P*<0.0083. No statistically significant relation at this significance level was found with positive affect scores, nor were there any significant associations with average total sleep time (linear and curvilinear term), sleep onset latency, and wake after sleep onset.

**Table 3:** Multiple regression of well-being by sleep variables measured by actigraphy.

	Combined sample*(N = 441)*			Predominantly White subsample *(n = 313)*			African American subsample *(n = 128)*		
	*β*	*t*	*P*	*β*	*t*	*P*	*β*	*t*	*P*
**Positive Well-being**									
*Satisfaction with Life Scale*									
Total Sleep Time (linear term)	−.08	−1.58	.115	−.10	−1.55	.122	−.02	−0.19	.852
Total Sleep Time (quadratic term)	.02	0.33	.739	.02	0.41	.680	−.01	−0.06	.955
Variability of Total Sleep Time	−.16	−3.01	.003	−.17	−2.63	.009	−.19	−1.83	.070
Wake After Sleep Onset	−.03	−0.65	.518	−.01	−0.13	.901	−.08	−0.85	.396
Sleep Onset Latency	−.02	−0.44	.658	.03	0.55	.583	−.08	−0.73	.465
*Positive Affect*									
Total Sleep Time (linear term)	−.02	−0.30	.761	−.05	−0.69	.492	.07	0.68	.496
Total Sleep Time (quadratic term)	.05	1.07	.287	.01	0.22	.830	.13	1.17	.243
Variability of Total Sleep Time	−.12	−2.08	.038	−.12	−1.73	.084	−.18	−1.69	.094
Wake After Sleep Onset	−.05	−1.02	.308	.04	0.70	.486^a^	−.22	−2.24	.027^a^
Sleep Onset Latency	.00	−0.04	.966	.05	0.71	.481	.00	0.05	.962
**Symptoms of Distress**									
*General Distress Depressive Symptoms*									
Total Sleep Time (linear term)	.10	1.74	.083	.04	0.66	.511	.16	1.52	.132
Total Sleep Time (quadratic term)	.03	0.66	.511	−.02	−0.35	.725^a^	.22	2.02	.046^a^
Variability of Total Sleep Time	.19	3.39	<.001	.17	2.66	.008	.23	2.08	.039
Wake After Sleep Onset	−.02	−0.36	.716	−.06	−1.01	.314	.02	0.20	.841
Sleep Onset Latency	.03	0.45	.650	−.02	−0.24	.814	.04	0.39	.695
*General Distress Anxious Symptoms*									
Total Sleep Time (linear term)	.14	2.43	.016	.07	1.17	.242	.20	1.87	.064
Total Sleep Time (quadratic term)	.00	0.08	.940	−.03	−0.47	.641	.09	0.82	.415
Variability of Total Sleep Time	.27	4.82	<.001	.27	4.27	<.001	.26	2.39	.019
Wake After Sleep Onset	−.02	−0.30	.767	.00	0.00	.999	−.04	−0.41	.685
Sleep Onset Latency	−.02	−0.40	.692	−.06	−1.01	.313	.01	0.08	.940
*Loss of Interest*									
Total Sleep Time (linear term)	.13	2.25	.025	.09	1.35	.178	.17	1.64	.105
Total Sleep Time (quadratic term)	−.04	−0.87	.383	−.03	−0.42	.673	−.01	−0.04	.966
Variability of Total Sleep Time	.16	2.88	.004	.15	2.29	.023	.21	1.92	.057
Wake After Sleep Onset	.00	0.05	.962	−.05	−0.86	.390	.04	0.39	.699
Sleep Onset Latency	.05	0.81	.419	−.01	−0.11	.913	.06	0.54	.590
*Anxious Arousal*									
Total Sleep Time (linear term)	.13	2.26	.024	.06	0.97	.331	.23	2.14	.034
Total Sleep Time (quadratic term)	.06	1.27	.206	.01	0.25	.805	.19	1.71	.090
Variability of Total Sleep Time	.19	3.43	<.001	.16	2.57	.011	.21	1.92	.057
Wake After Sleep Onset	.06	1.08	.283	.16	2.79	.006^a^	−.08	−0.78	.437^a^
Sleep Onset Latency	.04	0.75	.455	.07	1.12	.264	.05	0.50	.618

Note. Standardized regression coefficients are presented. Simultaneous entry of Total Sleep Time-linear term, variability of Total Sleep Time, Wake After Sleep Onset, and Sleep Onset Latency; Total Sleep Time-quadratic term was entered in separate models controlling Total Sleep Time-linear term, variability of Total Sleep Time, Wake After Sleep Onset, and Sleep Onset Latency. All analyses control age, gender, marital status, educational attainment, BMI, and twin status. Analyses of the combined sample additionally control sample membership (predominantly white American sample vs. urban African American sample).

Bonferroni-corrected significance level: *P*<.0083.

a coefficients are significantly different (*P*<.05) in the predominantly white American sample and in the urban African American sample.

Comparisons of the associations between the two subsamples show that there was a stronger negative relationship between wake after sleep onset and positive affect and a stronger curvilinear relationship between average total sleep time and the depressive symptoms scale (such that long and short sleep duration were predictive of higher symptom levels) in the urban African American sample (both “sample membership×sleep measure interaction terms” significant at *P*<0.05). These relations in the urban African American sample were, however, not significant at the Bonferroni-corrected significance level (P<0.0083). Moreover, there was a stronger positive relationship of wake after sleep onset with anxious arousal scores in the predominantly white American sample (*P* = 0.006).

**Figure 1:**
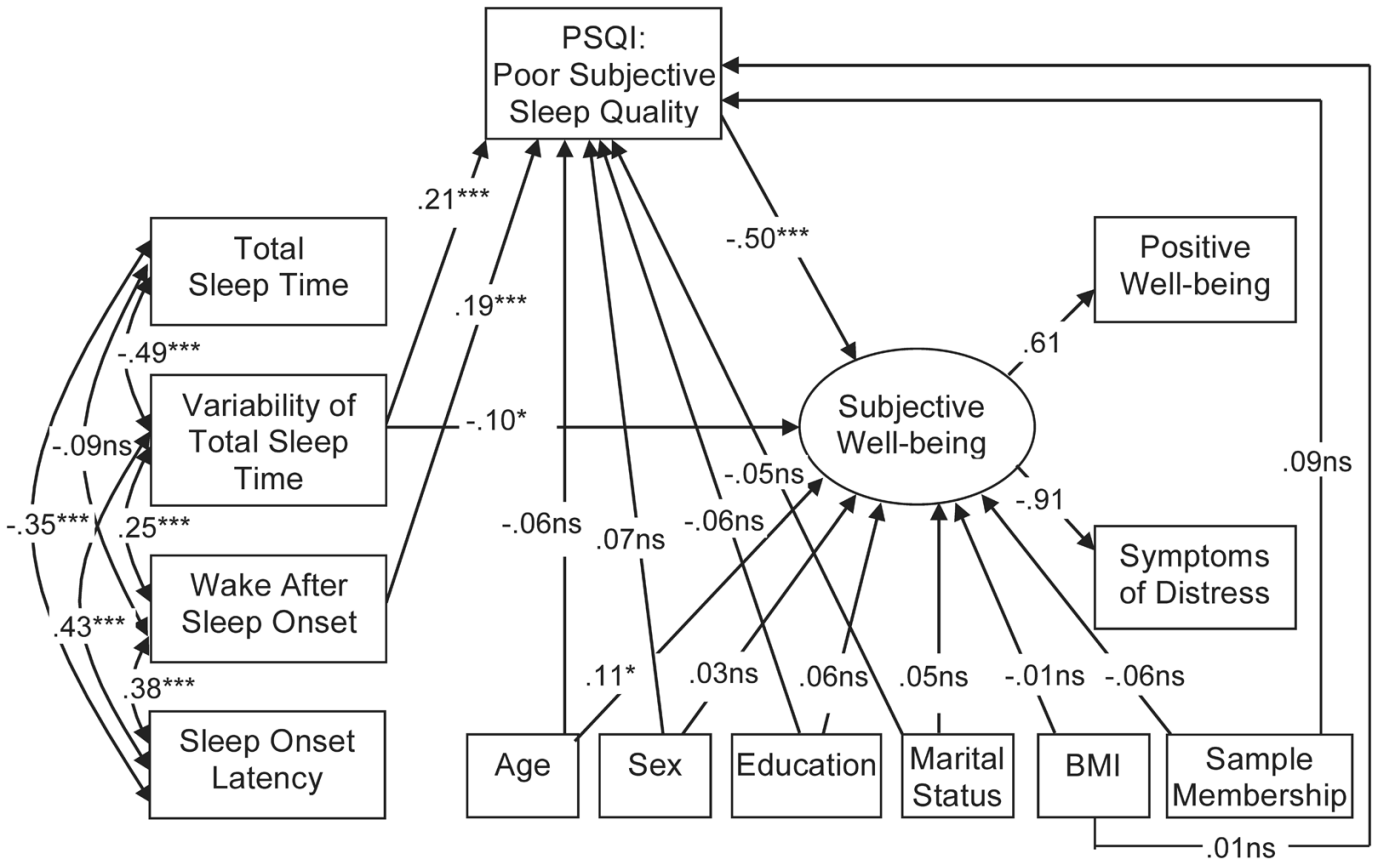
Relations of actigraphic sleep indices, subjective sleep quality, and subjective well-being. Standardized estimates are presented. *Chi^2^ = *32.9, *df = *16, *P = *0.008; *CFI = *0.98*; RMSEA = *.049; *GFI* = 0.99; *AGFI* = 0.94. Note. Total Sleep Time, Variability of Total Sleep Time, Wake After Sleep Onset, and Sleep Onset Latency were assessed by actigraphy. Non-significant paths from actigraphy measures were trimmed, non-significant paths from covariates were not trimmed. Abbreviations: PSQI: Pittsburgh Sleep Quality Index; BMI: Body Mass Index. *P<.05, **P<.01, ***P<.001, ^ns^ *P*>.05.

Figure 1 shows the structural equation model testing the relation between actigraphy sleep measures and subjective well-being with subjective sleep quality as mediator in the combined sample. The model in which non-significant paths were removed, showed an acceptable model fit (*Chi^2^* = 32.9, *df = *16, *P = *0.008, *CFI* = .98; *RMSEA* = .049; *GFI* = .99; *AGFI* = .94). Variability of total sleep time and wake after sleep onset were predictive of lower subjective sleep quality and, in turn, lower subjective sleep quality was predictive of lower subjective well-being. Indirect (mediating) effects of variability of total sleep time and wake after sleep onset on well-being through subjective sleep quality were significant (*β* = −.11 [95% *CI*: −.06 to −.15] *P = *0.002 and *β* = −.10 [95% *CI*: −.06 to −.14] *P = *0.002 for indirect effects of variability of total sleep time and wake after sleep onset, respectively). The direct and the indirect effect of variability of total sleep time on subjective well-being were approximately similar in strength. Total sleep time and sleep onset latency were not related to subjective sleep quality and subjective well-being.

## Discussion

The aim of the study was to examine the relationship between subjective well-being and sleep duration, variability of sleep duration, and sleep quality assessed with wrist actigraphy. Moreover, we tested whether this relationship was mediated by subjective sleep quality and whether it differed by race(/residential neighborhood. We found that higher variability of total sleep time was – among the actigraphy measures of sleep – the most consistent predictor of subjective well-being. This effect was partially mediated by subjective sleep quality and could be found in two diverse samples – a predominantly white American sample and an urban African American sample. Higher levels of wake after sleep onset were also indirectly related to lower levels of subjective well-being via subjective sleep quality. By contrast, mean total sleep time was neither directly nor indirectly related to subjective well-being and subjective sleep quality when controlling other sleep indices and covariates.

Our findings are consistent with studies showing that large intra-individual day-to-day variability of sleep is related to a combination of high stress levels and negative affect in adults [17], to higher levels of behavioral problems in adolescents [7], and to lower levels of subjective well-being and functional status in older demented individuals [34]. Moreover, the findings are consistent with evidence from diverse samples indicating that large variability of sleep duration is related to sleep problems [24]–[27], [32]. Also in line with earlier studies [15], [17], [18], we found that the commonly used average measures of actigraphy (total sleep time, wake after sleep onset, sleep onset latency) were not consistently related to measures of subjective well-being.

On a correlational level, average measures of objectively defined sleep quality as well as variability of sleep duration were modestly to moderately related to subjective sleep quality which is approximately similar to the size of relationship reported by Vitiello et al. [14]. This finding is, however, in contrast to other studies that did not observe a significant relationship between objective measures of sleep quality and subjective sleep quality [13], [15].

Our findings add to the body of evidence that intra-individual day-to-day variability of sleep duration is linked to both subjective sleep quality and subjective well-being. Studies on sleep patterns in insomnia patients have repeatedly pointed to the importance of regular sleep times (and – relatedly – to regularity of sleep duration) [25], [27], [31]. Individuals with insomnia are reported to have more variable sleep times than healthy people [24], [28], and it has been suggested that day-to-day variability in sleep times/sleep duration may be a mechanism that upholds symptoms of insomnia. Individuals who sleep poorly on one night may want to “catch up” by sleeping longer on the subsequent night. Although this recovery sleep may provide temporary improvements in feelings of fatigue and sleepiness, it can result in worse sleep the following night, as overly long sleep can undermine the ability to fall asleep [29], [30]. Consequently, poor sleep hygiene has been discussed as a causal mechanism that may trigger insomnia and eventually compromise well-being. To enable patients to have more regular sleep–wake patterns is therefore an integral part of behavioral treatment of insomnia [31]. Thus, one possible interpretation of our findings is that a highly variable sleep schedule may provoke sleep problems and poor subjective well-being.

Relatedly and pointing to the possible importance of taking also variability measures of well-being into account in future studies, a recent study [54] showed a relationship of variability in positive affect (as a marker of fluctuation in affective states) with average sleep efficiency assessed by actigraphy such that large day-to-day variability in positive affect was associated to poor sleep efficiency.

As a limitation of our study, the cross-sectional design precludes drawing firm conclusions regarding the causal direction of the relationship between variability of sleep duration and well-being. Based on the present findings it is equally possible that good subjective well-being has a favorable effect on sleep as vice versa. Moreover, based on our results it is not possible to resolve the question whether individual day-to-day variability of sleep duration is a consequence of (a voluntarily chosen or by practical/work related constraints imposed) irregularity of bed and rise times or whether it rather reflects a sleep disorder leading to a sequence of nights with short and poor sleep followed by nights with long recovery sleep evoked by homeostatic sleep pressure. Nevertheless, a strength of the study is that the associations could be replicated in two independent and diverse samples. While there were considerable mean level differences between the predominantly white American sample and the urban African American sample – which is in line with reports from other studies [17], [22], [36], [37], [38], the pattern of relations among the study variables were rather similar in both subsamples.

## Conclusion

The results of the present study support the common sense notion that a low day-to-day variability in sleep duration associates with better subjective sleep quality and higher levels of subjective well-being.

## Supporting Information

Table S1 **Detailed information on the procedure applied in model estimation.**
(DOCX)
