# Supplementary material for: Variability of Sleep Duration Is Related to Subjective Sleep Quality and Subjective Well-Being: An Actigraphy Study
Source: PLoS One. 2013 Aug 14;8(8):e71292. doi: 10.1371/journal.pone.0071292 (PMC3743871; doi:10.1371/journal.pone.0071292)
Supplement: Table S1 — Detailed information on the procedure applied in model estimation. (DOCX) [file pone.0071292.s001.docx]

Table S1. Detailed information on the procedure applied in model estimation.

|  | **Model** | **Description** | **Chi-Square** | **CFI** | **RMSEA** | **GFI** | **AGFI** |
| --- | --- | --- | --- | --- | --- | --- | --- |
| 1 | Independence Model | No relations between variables | Chi2=1070.7 df=91 P<0.001 | - | 0.156 | - | - |
| 2 | Saturated Model | All variables are allowed to correlate | Chi2=0 df=0 | - | - | - | - |
| 3 | Baseline Model on full dataset (FIML estimation of missing variables) | Theoretically derived model with the following rules: (A) All exogenous variables (actigraphy measures and control variables) are allowed to correlate. (B) Structural paths from all actigraphy measures on subjective well-being and subjective sleep quality. (C) Structural path from subjective sleep quality on well-being. (D) Structural paths from control variables on subjective sleep quality and subjective well-being | Chi2=24.7 df=11 P=0.010 | 0.99 | 0.053 | - | - |
| 4 | Baseline Model on correlation matrix (pairwise exclusion of missing variables) | Identical model specification as model 3. Estimation based on correlation matrix. | Chi2=25.2 df=11 P=0.008 | 0.99 | 0.054 | 0.99 | .93 |
| 5 | Trimmed Model (model which is presented in Figure 1) | Based on Baseline model (4) but with additional model trimming: (A) Constraining of non-significant structural paths from actigraphy measures on subjective well-being and subjective sleep quality to 0. (B) No trimming of structural paths from control variables on subjective sleep quality and subjective well-being. | Chi2=32.9 df=16 P=0.008 | 0.98 | 0.049 | 0.99 | .94 |

Note. Following the suggestions of Anderson and Gerbing (1988), first, a baseline model was tested (here: Model 3). The baseline model was theoretically derived and included paths from all actigraphy measures on subjective well-being (i.e., paths from the predictors on the outcome), paths from all actigraphy measures on subjective sleep quality (i.e., paths from the predictors on the mediator), and one path from subjective sleep quality on subjective well-being (i.e., path from the mediator on the outcome). The baseline model also included participant gender, age, marital status, education, BMI, and sample membership (predominantly white subsample vs. urban African American subsample) as control variables; therefore, paths from all control variables on mediator and the outcome were included. Moreover, all exogenous variables (the predictors and control variables) were allowed to correlate. The model was tested in the combined sample using sample membership as a control variable. To estimate missing data the full information maximum likelihood (FIML) method was applied by using the full SPSS data file as data source. In a second step, the model was tested again using the correlation matrix (with pairwise exclusion of missing data) as data source (Model 4), which has the advantage that bootstrapping procedures become possible. Inspection of the model showed that fit indices and parameter estimates were very similar as for Model 3; thus, Model 4 was used as starting point for further model specifications. In a third step, the model was trimmed such that non-significant paths were constrained to 0 (Model 5) (except for paths from control variables that also remained in the trimmed model if not significant). In Model 5, five paths were constrained. Model 5 is presented in Figure 1.
